# Extreme climate event promotes phenological mismatch between sexes in hibernating ground squirrels

**DOI:** 10.1038/s41598-021-01214-5

**Published:** 2021-11-04

**Authors:** Caila E. Kucheravy, Jane M. Waterman, Elaine A. C. dos Anjos, James F. Hare, Chris Enright, Charlene N. Berkvens

**Affiliations:** 1 Department of Biological Sciences, University of Manitoba, Winnipeg, MB R3T 2N2 Canada; 2 Assiniboine Park Zoo, 2595 Roblin Boulevard, Winnipeg, MB R3R 0B8 Canada

**Keywords:** Ecology, Zoology, Ecology

## Abstract

Hibernating ground squirrels rely on a short active period for breeding and mass accrual, and are thus vulnerable to extreme climate events that affect key periods in their annual cycle. Here, we document how a heatwave in March 2012 led to a phenological mismatch between sexes in Richardson’s ground squirrels (*Urocitellus richardsonii*). Females emerged from hibernation and commenced breeding earlier in 2012 relative to average female emergence. Although males had descended testes and pigmented scrota, it appeared that not all males were physiologically prepared to breed since 58.6% of males had non-motile sperm when breeding commenced. Body condition, relative testes size, and the relative size of accessory glands were significant predictors of sperm motility. Males with non-motile sperm had smaller accessory glands than males with motile sperm. There was no decrease in the number of juveniles that emerged in 2012 or female yearlings recruited in 2013, nor did juveniles emerge later than other years. The impact of this heatwave on male ground squirrels emphasizes the importance of assessing the consequences of climate change on the breeding success of hibernating species in both sexes, since the different sensitivity to external cues for emergence led to a mismatch in timing under this event.

## Introduction

Increasing temperatures and climate variability as a result of climate change have led to severe consequences for many species around the globe^1,2^. Shifts in phenology are a well-documented effect of climate change on organisms. Warming spring temperatures have led to advances in breeding dates, parturition and hatch dates, earlier emergence from hibernation, and earlier migration, among others^3–7^. While these phenological shifts have proven beneficial for some species, in terms of increased body mass and population growth due to earlier growing season onset, individuals of many species experience reduced fitness, reduced survival rate, with concomitant population declines, owing to asynchronies between breeding and food supply^4,5,8–11^.

Recently, a greater focus has been placed on how organisms are affected by stochastic weather events. The incidence of extreme events attributed to climate change has increased substantially since the 1950s^12^. Climate change is expected to further increase the frequency and magnitude of extreme climate events, such as temperature extremes (heat waves and cold spells), storms, droughts and fires^2,13^. The Intergovernmental Panel on Climate Change (IPCC) defines a climate extreme as “the occurrence of a value of a weather or climate variable above (or below) a threshold value near the upper or lower ends of the range of observed values of the variable”, depending on the location, time of year, and period of reference^13^. This definition is useful for categorizing extreme events in a climate-specific setting. In attempting to explore the biological effects of extreme climate events, however, it is becoming increasingly important to define extreme events in a more setting- and organism-specific context^14,15^. For example, Gutschick and BassiriRad^16^ define an extreme event as one that exceeds the acclimatory capacity of an organism, while Bailey and van de Pol^17^ consider an episode to be extreme when the climate or the conditions trigger a negative threshold-dependent biological response. In other words, once a climate event is deemed extreme, we must further consider the conditions of the event in terms of an organism’s biological capacity to tolerate it^15^. Extreme climate events have elicited various biological responses, including reduced body condition, shifts in species distributions, changes in functional community structure and community dynamics, and mass mortality events^14,18–20^. In some cases, extreme events can have a greater impact on species demography than long-term changes in climate averages^21^. Therefore, as climate change progresses, an organism’s ability to withstand extreme climate events and climate variability through physiological, behavioural and phenological adaptations will prove critical to their survival^22,23^.

Ground squirrels are vulnerable to both long-term changes in climate and extreme climate events. Many species of ground squirrel in North America are obligate hibernators and rely on a short active season for breeding and accrual of mass necessary to survive hibernation^24,25^. Endogenous circannual rhythms drive annual events in ground squirrels, such as immergence and emergence from hibernation^26^. Female emergence from hibernation and breeding is generally timed so that lactation coincides with low foraging costs^27^. However, females may be more flexible in emergence timing and appear to be more sensitive to environmental cues than males when emerging from hibernation, especially cues such as extended snow cover^28–30^. Therefore, female emergence can vary significantly depending on spring climate conditions^30–32^. Even with this apparent plasticity, female ground squirrels are sensitive to long-term climate trends and climate events that affect these key periods in their annual cycle^3,33^. For example, delayed spring snowmelt and reduced summer rainfall in Alberta, Canada has led to delayed emergence from hibernation and decreased mean annual fitness in female Columbian ground squirrels (*Urocitellus columbianus*)^34,35^. Years with prolonged snow cover resulted in reduced reproduction and weaning success in several species of marmot^36^. Similarly, late spring snowstorms have delayed female reproductive phenology in Columbian ground squirrels and Arctic ground squirrels (*Urocitellus parryii*)^37,38^. From these occurrences, it is evident that extreme climate events can affect the phenology and reproductive success of female ground squirrels.

Fewer studies have examined how such events affect male reproductive phenology, though some suggest that males demonstrate less plasticity in the timing of emergence than females during extreme or uncharacteristic weather^38^. In general, hibernating male ground squirrels appear to follow endogenous circannual cues for terminating torpor and emerging from hibernation more strictly, since endogenous rhythms trigger the circulation of testosterone, which prevents continued hibernation^24,26,29,39–41^. Although male ground squirrel emergence also varies among years, the hormonal activity following arousal from hibernation may hinder males’ ability to respond plastically to spring climate variation to the same degree females do, potentially resulting in a temporal mismatch between sexes prior to breeding^24,38,42^. Thus, spring climate events may also affect male ground squirrels’ reproductive success.

In Richardson’s ground squirrels (*Urocitellus richardsonii*), both male and female squirrels are typically reproductively mature as yearlings, though not all may breed^42^. After termination of torpor, males remain in hibernacula for 8.8 days on average during which they initiate testicular growth and maturation or recrudescence, augmentation of the accessory glands, and spermatogenesis, but do not necessarily complete spermatogenesis prior to emergence^29,43,44^. The development of accessory glands, including the prostate, seminal vesicles, and Cowper’s gland, lags slightly behind that of the testes^43^, but the contribution of accessory glands to seminal fluid is significant in mammals^45^. Males typically emerge in mid-March to establish a territory promoting optimal access to breeding opportunities and to gain mass prior to breeding^29,42^. Females emerge gradually from hibernation 8–16 days later and individuals usually mate within 4 days of emergence^46,47^. The breeding season is relatively short (3–4 weeks) but highly competitive. Most females breed within their first estrus cycle after emergence^46^. Females have only one litter per year but will mate with multiple males during estrus^48^, which results in multiple paternity within litters^49^. Therefore, the period of reproductive maturation prior to female emergence is essential for males to prepare for breeding, since males must be prepared to breed prior to female emergence or risk losing out on breeding opportunities^42^.

In March 2012 a heatwave spread across much of the eastern United States and central and eastern Canada, resulting in the warmest March on record across this region^50,51^. This extreme climate event led to significant crop losses (due to early blooming and subsequent frost) in the upper Midwest United States and Ontario, Quebec and New Brunswick, as well as flooding in several U.S. Gulf states, Quebec and New Brunswick^50,51^. However, little is documented about how this historic heatwave affected fauna, and particularly those species that may be sensitive to spring climate conditions as a cue for reproduction.

The objective of this study was to explore the impact of the March 2012 heatwave on the reproductive phenology of males in hibernating Richardson's ground squirrels. The majority of male squirrels were infertile in at the beginning of the breeding season in 2012, while few or no males were infertile in at the beginning of the breeding season in 2010 and 2014. We hypothesized that, due to unusually high temperatures, females would emerge earlier relative to males than in other years, and that the majority of males would not have sufficient time between termination of hibernation and the beginning of breeding to fully develop their reproductive organs. Males require time prior to female emergence to prepare for breeding^29^. We predicted that if females emerged before all males had fully developed, males with non-motile sperm would have smaller testes and/or smaller accessory glands than males with motile sperm.

## Materials and methods

### Specimen observation and collection

The Assiniboine Park Zoo (49˚52’N, 97˚14’W) in Winnipeg, Canada has a free-ranging Richardson’s ground squirrel population that has been the subject of intensive research exploring ground squirrel communication and cognitive abilities e.g. ^52^. Individuals within this population are routinely removed as part of a pest removal program^53^. Between 2007 and 2014, we recorded the date that the first male and female emerged from hibernation. Initial reports of emergence each year were provided by zoo staff, who were on site and monitoring activity daily. These initial reports were then confirmed via a site visit the same day and subsequent monitoring of population activity by J. Hare and students. The large size of the population, however, rendered recording the date of emergence for every individual impossible. Females were captured in live traps (Tomahawk Live Trap Co., WI; 13X13X40 cm, baited with peanut butter). Females were sexed upon first capture via visual inspection of external genitalia, and marked with numbered metal ear tags (Monel 1005; National Band & Tag Co., Newport, KY) and unique dye marks (Hydrience Pearl Black 52S; Clairol Inc., Stamford, CT) applied to their dorsal pelage. Female breeding dates were established by daily live-trapping of females after their emergence from hibernation, tracking their progression through estrus via visual examination of their urogenital papilla following criteria outlined by Murie and Harris^54^, and noting signs of breeding such as fresh semen and hardened copulatory plug material in the vagina, or dried semen caked in the hair surrounding a female's genitals. We recorded the number of females that successfully weaned litters and the number of female yearlings recruited (2008–2014), as well as the total number of female and male juveniles emerged and the dates of juvenile emergence (2007–2009, 2011–2013). In 2011, the number of female yearlings recruited was inferred based on weight at first capture after emergence since juveniles were not marked in 2010. We also recorded general observations of male mating behaviour.

Males were collected for the present study in 2010, 2012 and 2014. All males were collected opportunistically between first sighting of a male (12 March in 2010, 12 March in 2012 and 15 March in 2014) and the peak of the breeding season. Since males are removed as part of the Assiniboine Park Zoo pest removal program, collection ideally occurs before the peak of the breeding season. Males were collected by the authors with assistance from zoo staff. Since breeding commences as females emerge, collection depends on female rather than male emergence, so sampling dates are not standard among years and vary based on when the females emerge. We collected 24 males from 29 March-9 April in 2010, 29 males from 17–25 March in 2012, and 20 males from 28 March-5 April in 2014. The earliest emergence of females, reflecting the start of the breeding season, occurred on 20 March in 2010, 15 March in 2012, and 4 April in 2014. All collection was done with permission from Manitoba’s provincial wildlife agency, and collection and euthanasia methods were approved by the animal care committees of the University of Manitoba (F10-004, F10-030) and the Assiniboine Park Zoo (2012-A0001, 2014-A001) following American Society of Mammologists’ guidelines^55,56^ and ARRIVE guidelines.

We captured males in live traps and transported them to the zoo's veterinary clinic for euthanasia, where we followed the protocol described by Waterman et al.^53^. Males were identified by the presence of an external scrotum. We delivered 100% oxygen first in a chamber and then via a facemask at 2.0 L/min through an Isoflurane vaporizer set at 5% to anesthetize the animals. We then euthanized the animals with an intracardiac injection of potassium chloride. Attending veterinarians verified a deep plane of anesthesia through evaluation of palpebral and withdrawal reflexes, and confirmed individuals were deceased by the lack of a heartbeat upon thoracic auscultation and the complete loss of palpebral and corneal reflexes.

### Temperature

Temperature data for Winnipeg, Manitoba were downloaded from Environment and Natural Resources Canada (https://climate.weather.gc.ca/). The daily maximum temperature for March from 2010 to 2014 and historical temperature extremes for March were obtained primarily from station Winnipeg A CS (49°55' N, 97°14' W, years 1996–2020 available), although data were supplemented as necessary with data from station Winnipeg Richardson AWOS (49°55' N, 97°14' W, years 1953–2008 available) and station Winnipeg INTL A (49°54' N, 97°14' W, years 2008–2013 available). All three stations are located at the Winnipeg James Armstrong Richardson International Airport, which is located approximately 5 kms from the study site and experiences similar weather. We calculated a five-year (2010–2014) average maximum daily temperature for each day in March.

**Figure 1:**
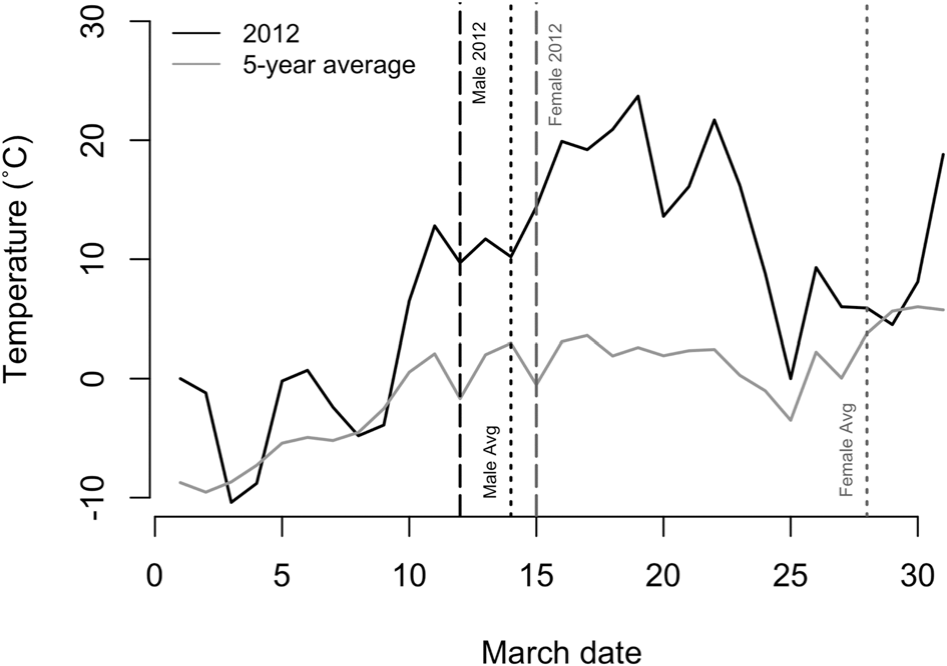
Daily maximum March temperatures (°C) in 2012 (black) compared to the 5-year average (2010–2014; grey) in Winnipeg, Canada. In 2012, male (black dashed line) Richardson’s ground squirrels (*Urocitellus richardsonii*) at the Assiniboine Park Zoo in Winnipeg, Canada began to emerge from hibernacula only 3 days before females (grey dashed line), compared to 5-year average male (black dotted line) and female (grey dotted line) emergence.

In 2012, temperatures up to 23.7 °C were experienced near the Assiniboine Park Zoo throughout the middle of March. Each day from 16 to 22 March recorded the highest temperature on record for those days. Around 10 March 2012 daily temperatures began to increase noticeably above the 5-yr average (Fig. 1). This spike coincided with the male and female Richardson’s ground squirrel emergence. Between 10 and 25 March, temperatures ranged from 3.5 °C (25 March) to 21.1 °C (18 March) above the 5-year average, for a mean of 12.9 °C above the 5-year average.

### Data collection and analysis

Following euthanasia, we measured body mass with a spring scale (Pesola, Baar, Switzerland) to ± 1.0 g and measured spine length from the occipital condyles to the base of the caudal vertebrae to ± 0.1 cm with a tape measure. Scrotum skin colour typically changes from a lighter, pinkish colour to darkly pigmented upon recrudescence when males are reproductively mature^57,58^. We visually classified scrotum colour as either pink or darkly-pigmented (dark gray or black). We removed the testes, epididymis and accessory glands, and used an MXX-212 Denver digital scale (Bohemia, NY, USA) to record the mass of the testes, prostate, seminal vesicles and Cowper’s gland (to ± 0.01 g). We cut the epididymis in a petri dish with warm coconut water^59,60^ and took a smear of sperm from the cauda epididymis within 5 min of euthanasia. We recorded whether spermatozoa were present and motile under a microscope (40X, Model CX41, Olympus American Inc^©^., USA). For all three years of study, sperm was classified as “motile” if spermatozoa were present and moving, and “non-motile” if not present or present and non-moving.

To assess the impact of the change in female phenology on male reproductive condition, we first combined the males with motile sperm and those with non-motile sperm across the three years of observation and constructed a logistic regression to assess the importance of body condition, relative testes, prostate, seminal vesicle, and Cowper’s gland size, and date relative to female emergence. We used the residuals of body mass regressed on spine length to assess body condition^61^. To account for differences in body size, we calculated relative size of the accessory glands by dividing testes mass, prostate mass, seminal vesicle mass, and Cowper’s gland mass by total body mass. Testes mass was missing from one male in 2014, and Cowper’s mass was missing from 2 males in 2012 and 6 males in 2014. Because males must be prepared to mate upon the emergence of the first females and testes size declines over the breeding season^44^, we used the date of individual male capture relative to emergence of the first female as a covariate. Due to missing observations in testes and Cowper’s gland size, we used 64 males for the analysis. We first calculated Pearson's correlation coefficients to explore correlations among the factors. To avoid multicollinearity, we scaled the data for the three accessory glands and performed a principal component analysis (PCA) among the three variables, using the first principal component as a factor in the logistic regression. We then used a two-way analysis of variance ANOVA with interaction to test for differences in body mass, testes mass, and accessory gland mass between males with non-motile sperm and males with motile sperm among the three years of study. Since the interaction term was not significant for body mass or testes mass, it was removed. All data were tested for homogeneity of variance and residuals for normality. We analyzed all data using RStudio version 1.1.463 with *p* ≤ 0.05 considered statistically significant.

## Results

### Emergence and breeding

Between 2007 and 2014, the first male emerged from hibernation between 12 and 21 March, while the first female emerged between 15 March and 12 April, on average 14.0 days (95% C.I.; 8.2–19.8 days) after males (Fig. 1). In 2012, females began to emerge early (15 March), only 3 days after males (12 March), who emerged near the average emergence date for males (Fig. 1). Males were actively courting females during the time following female emergence and before capture. The proportion of females that successfully weaned litters between 2008 and 2014 varied between 0.60 and 0.87 (Table 1). The number of female yearlings recruited into the population varied between 12 and 53 (mean ± SE = 29.9 ± 4.4), and the number of male and female juveniles at emergence varied between 114 and 375 (240.9 ± 37.9; Table 1). Juveniles emerged over a span of 11–20 days, typically from mid-May or early June to early or mid-June (Table 1).

**Table 1:** The proportion of female Richardson’s ground squirrels (*Urocitellus richardsonii*) that successfully weaned litters (the number of females that successfully weaned litters divided by the number of females that mated), the number of male and female juveniles emerged at the Assiniboine Park Zoo in Winnipeg, the dates of juvenile emergence, and the number of female yearlings recruited into the population, Canada between 2007 and 2014.

Year	Proportion that successfully weaned	Juvenile emergence dates	Juveniles emerged	Yearlings recruited
2007	–	16 May–5 June	114	34
2008	0.87	21 May–10 June	347	36
2009	0.79	5 June–16 June	153	19
2010	–	–	–	22
2011	0.60	1 June–21 June	170	29*
2012	0.82	16 May–4 June	238	12
2013	0.79	1 June–21 June	289	34
2014	0.81	1 June–16 June	375	53

*Number of yearling females inferred based on body weight at first capture since juveniles were not marked in the previous year.

### Analysis of sperm and scrota

Seventeen males in 2012 (58.6%, n = 29) and three males in 2014 (15.0%, n = 20) had sperm classified as non-motile, while no males in 2010 (n = 24) had sperm classified as non-motile. In 2012, there were three males with a pink scrotum (10.3%; n = 1 with motile sperm, n = 2 with non-motile sperm) and 26 males with a darkly-pigmented scrotum (89.7%; n = 11 with motile sperm, n = 15 with non-motile sperm). In 2010 and 2014 all males had a darkly-pigmented scrotum.

### PCA and logistic regression

Relative prostate, seminal vesicles, and Cowper’s gland mass were highly correlated (prostate-seminal vesicles, r = 0.762; prostate-Cowper’s gland, r = 0.775; seminal vesicles-Cowper’s gland, r = 0.754). No other factors were highly correlated (r ≤ 0.477). In the PCA, the component loadings for PC1 were in a uniform direction. PC1 accounted for 84.3% of the variance (eigenvalue = 2.527). We used PC1 in the logistic regression to represent the influence of the three accessory glands on motile vs. non-motile sperm production. For the three years of study, body condition (*β* = -0.05, *z* = − 2.152, *p* = 0.031, relative testes size (*β* = 1328.85*, z* = 1.993, *p* = 0.046), and PC1 (*β* = − 3.22, *z* = − 3.26, *p* = 0.001) were all significant predictors of sperm quality, while relative date was not significant (*β* = 0.13*, z* = 1.544, *p* = 0.12).

### Comparison between males with motile and non-motile sperm

**Figure 2:**
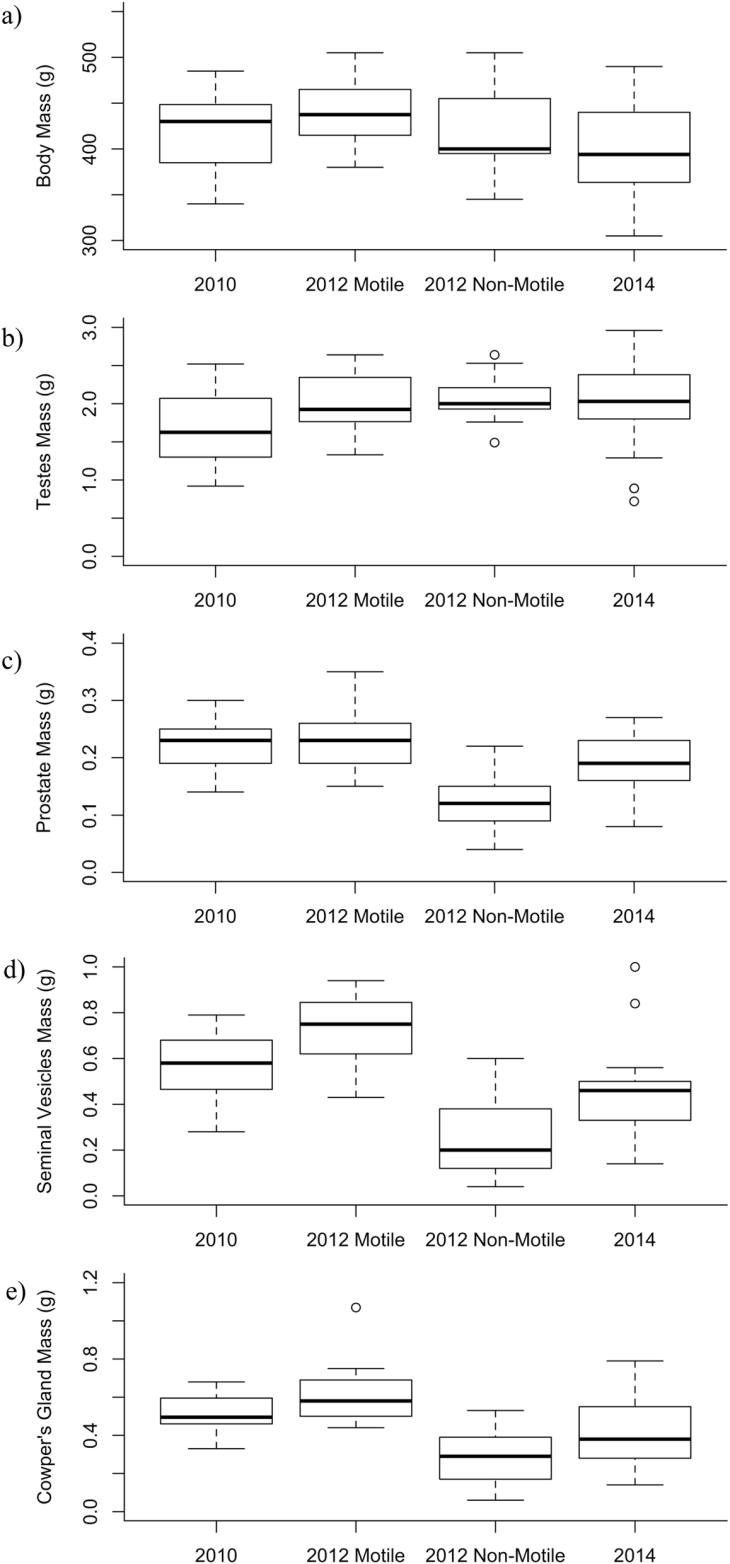
Male Richardson’s ground squirrels (*Urocitellus richardsonii*) at the Assiniboine Park Zoo in Winnipeg, Canada were analyzed for differences among years (2010, 2012, and 2014) and between those with motile sperm and those with non-motile sperm in 2012. We measured body mass (**a**), testes mass (**b**), prostate mass (**c**), seminal vesicles mass (**d**), Cowper’s gland mass (**e**).

There was no difference in body mass between males with motile sperm and males with non-motile sperm (two-way ANOVA; *F*_1,69_ = 0.02, *p* = 0.90), but there was a significant difference among years (two-way ANOVA; *F*_2,69_ = 3.31, *p* = 0.042; Fig. 2a). There was no difference in testes mass between males with motile sperm and males with non-motile sperm (two-way ANOVA; sperm: *F*_1,68_ = 1.53, *p* = 0.22) or among years (*F*_2,68_ = 2.93, *p* = 0.06; Fig. 2b). Males with motile sperm had greater accessory gland mass than males with non-motile sperm as well as a significant interaction term between sperm and year for prostate mass (two-way ANOVA; sperm: *F*_1,68_ = 47.35, *p* < 0.001, η^2^ = 0.38; interaction: *F*_1,68_ = 5.71, *p* = 0.020, η^2^ = 0.05; Fig. 2c), seminal vesicles mass (two-way ANOVA; sperm: *F*_1,68_ = 34.43, *p* < 0.001, η^2^ = 0.27; interaction: *F*_1,68_ = 18.17, *p* < 0.001, η^2^ = 0.14; Fig. 2d), and Cowper’s gland mass (two-way ANOVA; sperm: *F*_1,60_ = 29.44, *p* < 0.001, η^2^ = 0.29; interaction: *F*_1,60_ = 8.44, *p* = 0.005, η^2^ = 0.08; Fig. 2e). There was no significant difference among years in prostate mass (two-way ANOVA, year: *F*_2,68_ = 1.05, *p* = 0.35; Fig. 2c) or Cowper’s gland mass (two-way ANOVA, year: *F*_2,60_ = 1.87, *p* = 0.16; Fig. 2e), but there was a significant difference among years in seminal vesicle mass (two-way ANOVA, year: *F*_2,68_ = 3.23, *p* = 0.046, η^2^ = 0.05; Fig. 2d).

## Discussion

The March 2012 heatwave in Winnipeg, Manitoba can be considered extreme by both climatological and biological criteria. The heatwave could be considered biologically extreme for male Richardson’s ground squirrels by both Gutschick and BassiriRad’s^16^ and Bailey and van de Pol’s^17^ definitions of a biological extreme climate event, since females, who exhibit a greater amount of phenological plasticity in emergence in response to external conditions^31,38^, emerged from hibernacula approximately 13 days earlier than usual at this site and only 3 days after males. Males generally demonstrate less plasticity in emergence^26,38^ and in 2012 they began to emerge at approximately the same time as other years. This difference in timing generated indirect consequences for male reproduction, since in this study many males did not appear to be physiologically prepared for the early presence of females as the majority had non-motile sperm. Although we do not have data on pre-emergence euthermy for males in this population, it appears that females emerged prior to the average number of days required for gonadal development by males in 2012, and many males were not reproductively prepared for breeding. Therefore, the heatwave led to a phenological mismatch between male and female Richardson’s ground squirrels.

Despite the relatively low number of fertile males during the 2012 breeding season, there were no evident population-level effects of this event. The total number of juveniles that emerged in 2012 and the number of female yearlings recruited in 2013 did not decrease compared to previous years. Further, juvenile emergence, which began mid-May in 2012, was on the earlier end of the range seen throughout the years, suggesting that females did not take longer to become pregnant despite the proportion of males with non-motile sperm at the start of the breeding season. These results suggest that the consequences of the shift in phenology on male fertility did not have significant population-level effects in the current and subsequent breeding season, likely owing to multiple mating by females with fertile males in the population. Female Richardson’s ground squirrels will frequently mate with two or more males during estrus, often resulting in multiple paternity within litters^48,49^. One proposed hypothesis for the benefits of multiple mating in females is that it increases the probability of fertilization in the case of insufficient or infertile sperm^62^. Our study supports this hypothesis, since a similar number of females in this population were able to successfully wean litters as other years, despite over half of the males producing non-motile sperm. Although there was no effect on number of juveniles emerged in 2012 or yearlings recruited in 2013, the effective population size may have been reduced as a consequence of fewer males siring offspring^63^. While multiple mating may have buffered the impact of male infertility in 2012, long-term effects on the population remain unknown.

The reproductive and fitness consequences of shifts in phenology due to climate variables have been demonstrated in female Columbian ground squirrels^34,35,37^, but the impact of climate change on males may be more cryptic than in females. In females, reproductive status and success can be determined relatively easily by the condition of the vulva^42^ and subsequent pregnancy, parturition and weaning. Males are assumed to be in reproductive status when the scrotum is darkly pigmented and testes are fully recrudesced^42,64,65^. In 2012, only three males had a pink scrotum and there was no significant difference in body mass or testes mass between males with motile sperm and males with non-motile sperm. From these external indicators, there was no visible difference between the two groups to indicate infertility. To assess how climate change is impacting male hibernators, it may be necessary to look further to body condition and the reproductive organs.

Both body condition and relative testes size were significant predictors of sperm motility in our logistic regression model, though without individual emergence dates it is difficult to determine the exact role of body condition. In this study, males were sampled earlier than in other years due to the early emergence of females. It is possible that males who emerged earlier had more time to accrue mass and develop testes prior to female emergence than later-emerging males. Our results suggest that male body condition and relative testes size influenced this population’s ability to produce motile sperm under unusual conditions, likely due to differences in mass accrual between earlier- and later-emerging males. In future studies, detailed data of individual emergence date would help distinguish the role of body condition in reproductive success under extreme climate events.

The mass of the accessory reproductive glands (the prostate, seminal vesicles, and Cowper’s gland) were smaller in males with non-motile sperm, supporting the hypothesis that these males did not have sufficient time before breeding began to develop their reproductive organs after returning to homeothermy. There was no difference in prostate or Cowper’s gland mass among years and a significant interaction between sperm and year, suggesting that the primary reason for smaller glands was how the shift in female phenology in 2012 was timed relative to male reproductive development. Although there was a difference in seminal vesicles mass among years, this difference may be reflective of variation in body mass. Further, the effect size of sperm and the interaction term between sperm and year was much greater, supporting the conclusion drawn for the other two accessory glands.

Enlargement of the accessory glands and spermatogenesis lags behind the enlargement of the testes in sciurids, and hibernating male ground squirrels require 2–4 weeks of euthermic body temperatures to produce motile spermatozoa^39,43,66^. Seminal vesicles, prostate, and Cowper’s gland reached maximum size several weeks after testes reached maximum size in male thirteen-lined ground squirrels (*Ictidomys tridecemlineatus*)^43^, and seminal vesicles continued to increase in weight for several days following emergence in Belding’s ground squirrel (*Urocitellus beldingi*), while testes were at maximum size upon emergence^65^. In an Alberta population of Richardson’s ground squirrels, peak testis length was reached 11 days after emergence^42^. Spermatozoa are often absent in male Richardson’s ground squirrel seminiferous tubules upon emergence^44^. Michener^29^ found that males that had been euthermic for > 11 days had spermatozoa present in the seminiferous tubules, while males who had been euthermic for < 4 days had primary spermatocytes and spermatids, implying that spermatogenesis required at least 4 days of euthermy. In the Assiniboine Park Zoo population in 2012, the first males who emerged had only 3 days before the first females emerged, fewer than the minimum number of days specified by Michener^29^ for both spermatogenesis and testes maturation. Males with non-motile sperm had smaller accessory glands, suggesting that annual reproductive development and spermatogenesis was not completed for those individuals upon the commencement of breeding.

Given that climate change is predicted to increase the frequency and magnitude of extreme climate events, including heat waves^2,13^, male hibernators may face increasing pressure to adjust their phenology in response to both climatic conditions and shifts in breeding opportunities. Male and female breeding phenology is timed not only to the other sex, but also to seasonal changes and food abundance^3^. Any disturbance to this timing can impact an individual’s reproductive success, as predicted under the ecological mismatch hypothesis^8^. Other factors such as body condition may also influence an individual’s ability to cope with these pressures. Although the effects of shifts in phenology may not be as immediately evident in males as in females, there could be important implications for population dynamics. Although we did not discover any population-level consequences of the heatwave, future studies may explore the resilience of hibernating species’ reproductive output as extreme events become more frequent. It is critical to explore the consequences of climate change on both male and female reproduction, particularly in species who rely on a single, short breeding season each year.

## Data Availability

The datasets generated during and/or analysed during the current study are available from the corresponding author on reasonable request.
